# Supplementary material for: TRPV1 controls innate immunity during Citrobacter rodentium enteric infection
Source: bioRxiv. 2023 Jul 27:2023.07.26.550772. Preprint. [Version 1] doi: 10.1101/2023.07.26.550772 (PMC10402119; doi:10.1101/2023.07.26.550772)

bioRxiv preprint doi: https://doi.org/10.1101/2023.07.26.550772; this version posted July 27, 2023. The copyright holder for this preprint (which was not certified by peer review) is the author/funder, who has granted bioRxiv a license to display the preprint in perpetuity. It is made available under aCC-BY 4.0 International license.

Table S1. List of primers used for qPCR

**Target**	**Forward 5’-3’**	**Reverse 5’-3’**
Il1β	CTGTGACTCATGGGATGATGATG	CGGAGCCTGTAGTGCAGTTG
Il6	TAGTCCTTCCTACCCCAATTTCC	TTGGTCCTTAGCCACTCCTTC
Il17a	TTTAACTCCCTTGGCGCAAAA	CTTTCCCTCCGCATTGACAC
Il22	ATGAGTTTTTCCCTTATGGGGAC	CTGGAAGTTGGACACCTCAA
Ifnγ	GCCACGGCACAGTCATTGA	TGCTGATGGCCTGATTGTCTT
Tnfa	CCCTCACACTCAGATCATCTTCT	GCTACGACGTGGGCTACAG
Nos2	GTTCTCAGCCCAACAATACAAGA’	GTGGACGGGTCGATGTCAC
RegIIIγ	CCTCAGGACATCTTGTGTC	TCCACCTCTGTTGGGTTCA
Icam1	GTGATGCTCAGGTATCCATCCA	CACAGTTCTCAAAGCACAGCG
Vcam1	AGTTGGGGATTCGGTTGTTCT	CCCCTCATTCCTTACCACCC
Madcam1	CCTGGCCCTAGTACCCTACC	CCGTACAGAGAGGATACTGCTG
Cxcr2	ATGCCCTCTATTCTGCCAGAT	GTGCTCCGGTTGTATAAGATGAC
Cxcl1	TCCAGAGCTTGAAGGTGTTGCC	AACCAAGGGAGCTTCAGGGTCA
Cxcl2	CTCTCAAGGGCGGTCAAAAAGTT	TCAGACAGCGAGGCACATCAGGTA

bioRxiv preprint doi: https://doi.org/10.1101/2023.07.26.550772; this version posted July 27, 2023. The copyright holder for this preprint (which was not certified by peer review) is the author/funder, who has granted bioRxiv a license to display the preprint in perpetuity. It is made available under aCC-BY 4.0 International license.

Cxcl3	CATCCAGAGCTTGACGGTGA	ACACATCCAGACACCGTTGG
Cxcl6	TGGATCCAGAAGCTCCTGTGA	TGCATTCCGCTTAGCTTTCTTT
β-actin	GGCTGTATTCCCCTCCATCG	CCAGTTGGTAACAATGCCATGT

Table S2. Antibodies used for Confocal Imaging

**Target**	**Host**	**Source**	**Catalog No**	**Dilution**	
CD3	Rat	Bio-Rad	CD3-12	1:200	
βIII Tubulin	Mouse	ThermoFisher	MA1-118	1:5000	

bioRxiv preprint doi: https://doi.org/10.1101/2023.07.26.550772; this version posted July 27, 2023. The copyright holder for this preprint (which was not certified by peer review) is the author/funder, who has granted bioRxiv a license to display the preprint in perpetuity. It is made available under aCC-BY 4.0 International license.

CDH1	Mouse	ECM Bioscience	CP1921	1:300	
Ki67	Rabbit	LSBio	LS-C141898	1:600	
TRPV1	Rabbit	Alomone Labs	ACC-030	1:200	
**Target**	**Host**	**Conjugate**	**Source**	**Catalog No**	**Dilution**
Streptavidin		Alexa Fluor 488	ThermoFisher	S32354	1:200
Anti-rabbit	Goat	Alexa Fluor 488	ThermoFisher	A32732	1:200
Anti-rat	Donkey	Alexa Fluor 647	Abcam	Ab150155	1:200

bioRxiv preprint doi: https://doi.org/10.1101/2023.07.26.550772; this version posted July 27, 2023. The copyright holder for this preprint (which was not certified by peer review) is the author/funder, who has granted bioRxiv a license to display the preprint in perpetuity. It is made available under aCC-BY 4.0 International license.

Table S3: Flow cytometry antibodies

bioRxiv preprint doi: https://doi.org/10.1101/2023.07.26.550772; this version posted July 27, 2023. The copyright holder for this preprint (which was not certified by peer review) is the author/funder, who has granted bioRxiv a license to display the preprint in perpetuity. It is made available under aCC-BY 4.0 International license.

Antigen Target	Manufacture & Clone	Catalog number
CD3	Tonbo Biosciences 145-2C11	20-0031
CD4	BD Biosciences RM4-5	553047
CD45	Invitrogen 30-F11	48-0451-82
Ly6G	Tonbo Biosciences 1A8	35-1276
CD11b	Invitrogen M1/70	56-0112-82
IFNγ	Invitrogen XMG1.2	25-7311-82
IL-17A	BD Biosciences TC11-18H10	561020
IL-22	Invitrogen 1HBPWSR	46-7221-82
CD31	Biolegend MEC13.3	102533
gp38	Biolegend 8.1.1	127405
ICAM-1	Biolegend YN1/1.7.4	116114
VCAM-1	Biolegend 429(MVCAM.A)	105719
MAdCAM-1	Biolegend MECA-367	120710
Fixable Live/Dead Aqua	ThermoFisher	L34957
CD115	BD Bioscience T38-320	743638
SiglecF	BD Biosciences E50-2440	740557
Ly6C	Invitrogen HK1.4	25-5932-82
CXCR4	Miltenyi Biotec REA107	130-118-682
CXCR2	Miltenyi Biotec REA942	130-115-635
CD64	Miltenyi Biotech REA286	130-118-684
CD11c	Invitrogen N418	17-0114-81

bioRxiv preprint doi: https://doi.org/10.1101/2023.07.26.550772; this version posted July 27, 2023. The copyright holder for this preprint (which was not certified by peer review) is the author/funder, who has granted bioRxiv a license to display the preprint in perpetuity. It is made available under aCC-BY 4.0 International license.

Fig. S1 TRPV1 expressed in the colon does not affect gut permeability or epithelial cell proliferation. (A) Paraffin embedded colonic tissue of wild-type (WT) and TRPV1$^{-/-}$ mice was stained with anti-TRPV1 (red), anti-βIII-tubulin (green), and DAPI (blue). **(B)** WT (open circles) and TRPV1$^{-/-}$ (black circles) mice colons were assessed for gut permeability through Ussing chambers. Not significant. Student t test with 7-9 animals per group. **(C)** Proliferation of colon-derived organoids was assessed *in vitro* with EdU incorporation and diameter measurements. WT and TRPV1$^{-/-}$ derived organoids were compared. Not significant. Student t test comparing WT to TRPV1$^{-/-}$ with 7-11 organoids from 3 mice per group.

Fig. S2 IFNγ, IL-17A, and IL-22 show no difference in mRNA transcripts at baseline and 10- and 29- days post-infection by *C. rodentium*. (A-C) Colonic tissue from wild-type (WT) and TRPV1$^{-/-}$ mice was assessed by qPCR for expression of common T cell produced cytokines relevant to *C. rodentium* clearance such as **(A)** *Ifnγ*, **(B)** *Il17a*, and **(C)** *Il22*. Data are presented

bioRxiv preprint doi: https://doi.org/10.1101/2023.07.26.550772; this version posted July 27, 2023. The copyright holder for this preprint (which was not certified by peer review) is the author/funder, who has granted bioRxiv a license to display the preprint in perpetuity. It is made available under aCC-BY 4.0 International license.

as mean ± standard error of the mean: *, $P < 0.05$, **, $P < 0.01$ and ***, $P < 0.001$; one-way ANOVA with post-hoc analysis using Tukey's multiple comparisons test. 6–12 animals per group. **(D & E)** WT or TRPV1$^{-/-}$ negatively selected CD3^{+} CD4^{+} T cells were stained with CellProliferation dye eFluor450 and cultured *in vitro* at described concentrations of anti-CD3ε and anti-CD28 antibodies for 72 hours and then analyzed by flow cytometry for proliferation index. **(D)** Comparison of WT and TRPV1$^{-/-}$ T cell proliferation at different concentrations of anti-CD3ε and anti-CD28 antibodies. **(E)** WT T cells were cultured with 1 µg/mL of anti-CD3ε and 1 µg/mL of anti-CD28 and a dose response of the TRPV1 agonist capsaicin. After 72 hours, cells were analyzed by flow cytometry for proliferation index. Data are presented as mean ± standard error of the mean: not significant.

Fig. S3 Lamina propria T cell gating strategy. Whole colon dissociated into a single cell suspension and stained for antibodies to identify live CD45^{+} CD3^{+} CD4^{+} T cells.

Fig. S4 TRPV1 deletion did not alter neutrophil precursors in the bone marrow compartment. Wild-type (WT) and TRPV1$^{-/-}$ mice had right femur bone marrow extracted at baseline and 10 days p.i. of *C. rodentium* and stained for pre-neutrophils (SiglecF^{-}, CD115^{-}, Gr-1^{+}, CD11b^{+}, CXCR4^{+}), immature neutrophils (SiglecF^{-}, CD115^{-}, Gr-1^{+}, CD11b^{+}, CXCR4^{-}, CXCR2^{-}), and mature neutrophils (SiglecF^{-}, CD115^{-}, Gr-1^{+}, CD11b^{+}, CXCR4^{-}, CXCR2^{+}, Ly6G^{+}). **(A)** Bone marrow gating strategy and **(B)** frequency of live of each subset of neutrophil lineage shown. Data are presented as mean ± standard error of the mean: one-way ANOVA with Tukey post-test, with 7–12 animals per group, not significant. **(C)** Gating strategy for whole colon dissociated into a single cell suspension and stained for antibodies to identify live (CD45^{+}, Ly6G^{+}, CD11b^{+}) neutrophils, (CD45^{+}, Ly6G^{-}, Ly6C^{+}) monocytes, (CD45^{+}, Ly6G^{-}, Ly6C^{-}, CD64^{+}) macrophages, (CD45^{+}, Ly6G^{-}, Ly6C^{-}, CD64^{-}, CD11c^{hi}) conventional dendritic cells (DC).

bioRxiv preprint doi: https://doi.org/10.1101/2023.07.26.550772; this version posted July 27, 2023. The copyright holder for this preprint (which was not certified by peer review) is the author/funder, who has granted bioRxiv a license to display the preprint in perpetuity. It is made available under aCC-BY 4.0 International license.

Fig. S1

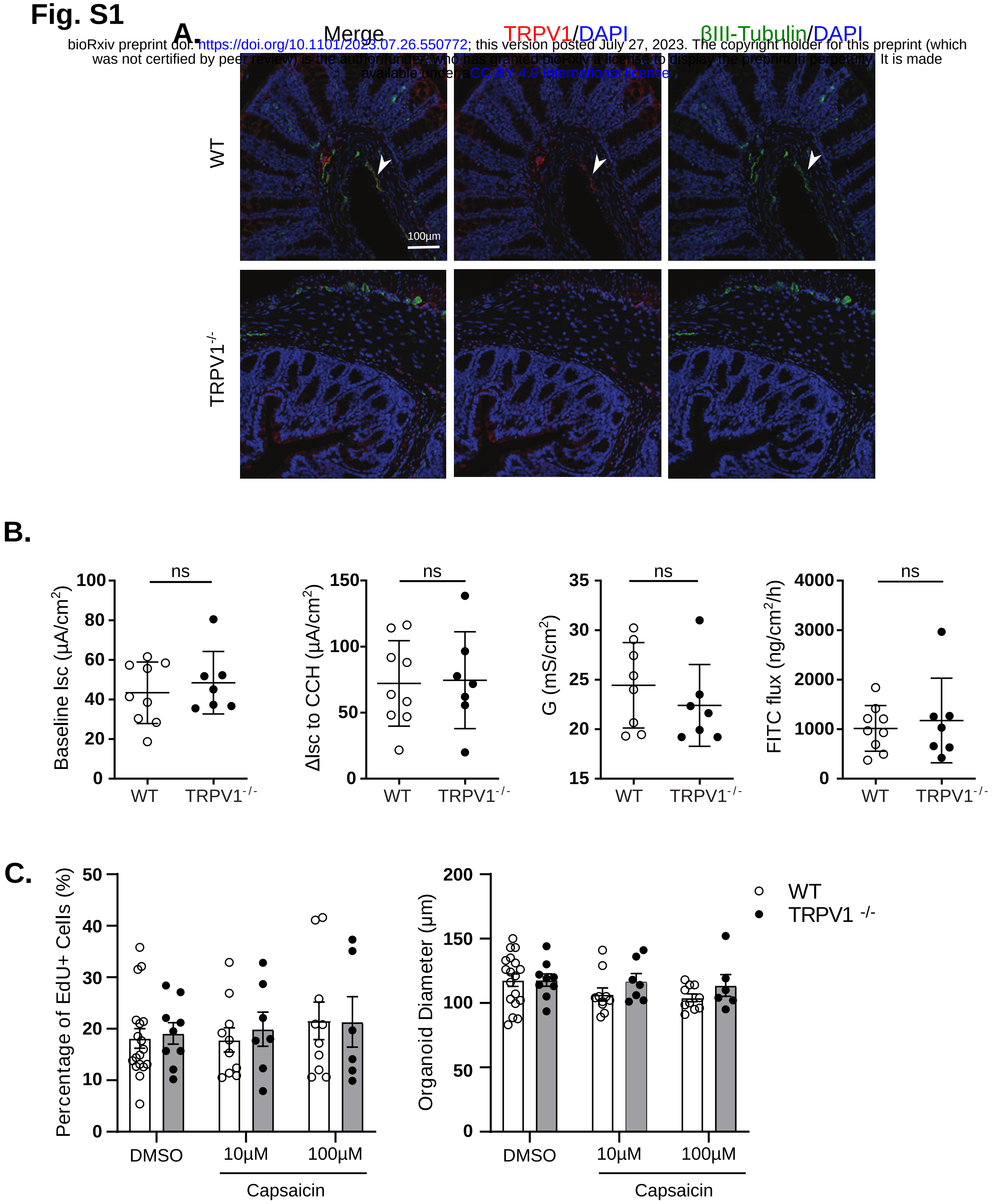

bioRxiv preprint doi: https://doi.org/10.1101/2023.07.26.550772; this version posted July 27, 2023. The copyright holder for this preprint (which was not certified by peer review) is the author/funder, who has granted bioRxiv a license to display the preprint in perpetuity. It is made available under aCC-BY 4.0 International license.

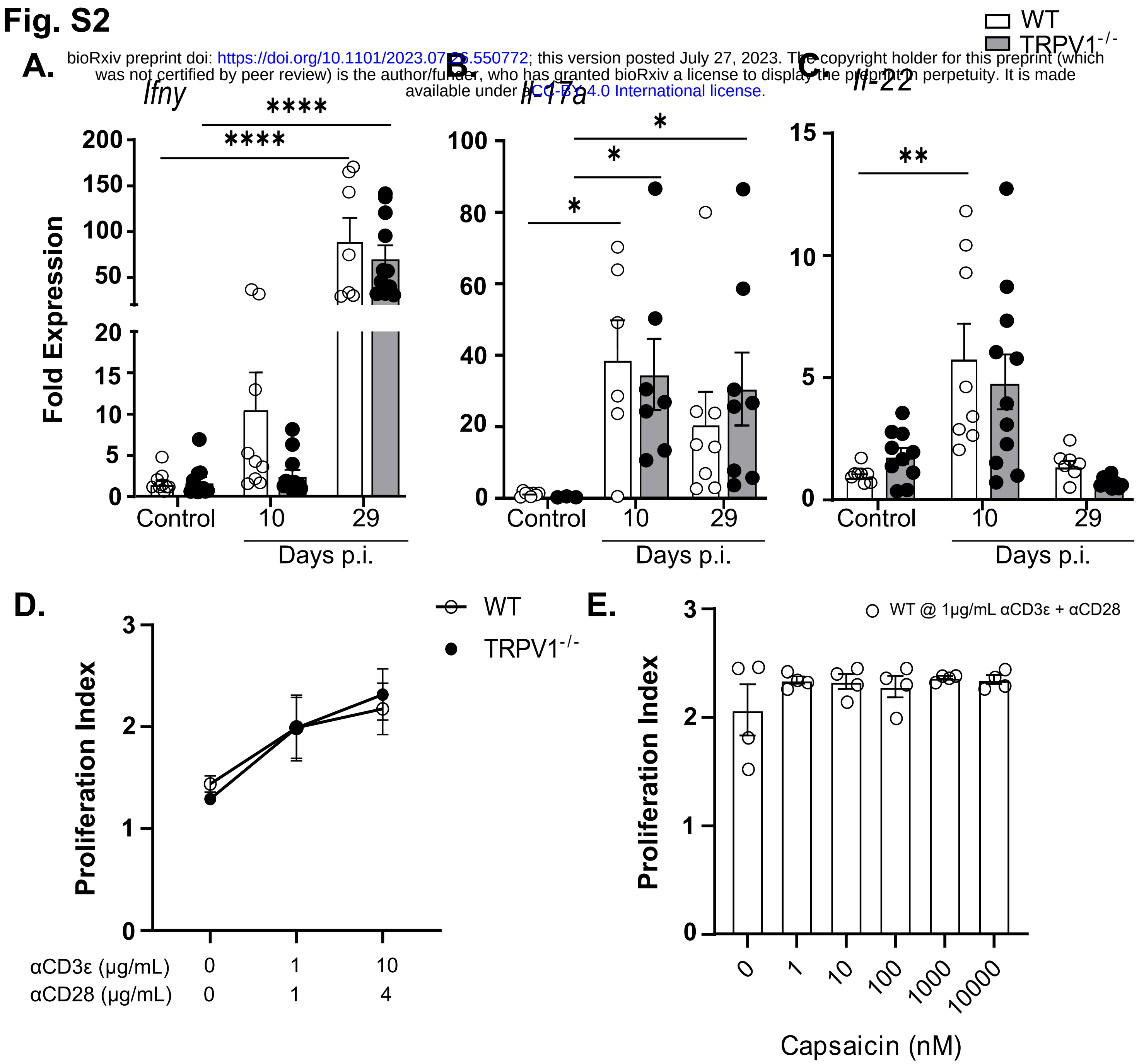

Fig. S3

bioRxiv preprint doi: https://doi.org/10.1101/2023.07.26.550772; this version posted July 27, 2023. The copyright holder for this preprint (which was not certified by peer review) is the author/funder, who has granted bioRxiv a license to display the preprint in perpetuity. It is made available under aCC-BY 4.0 International license.

Lamina Propria T Cell Gating

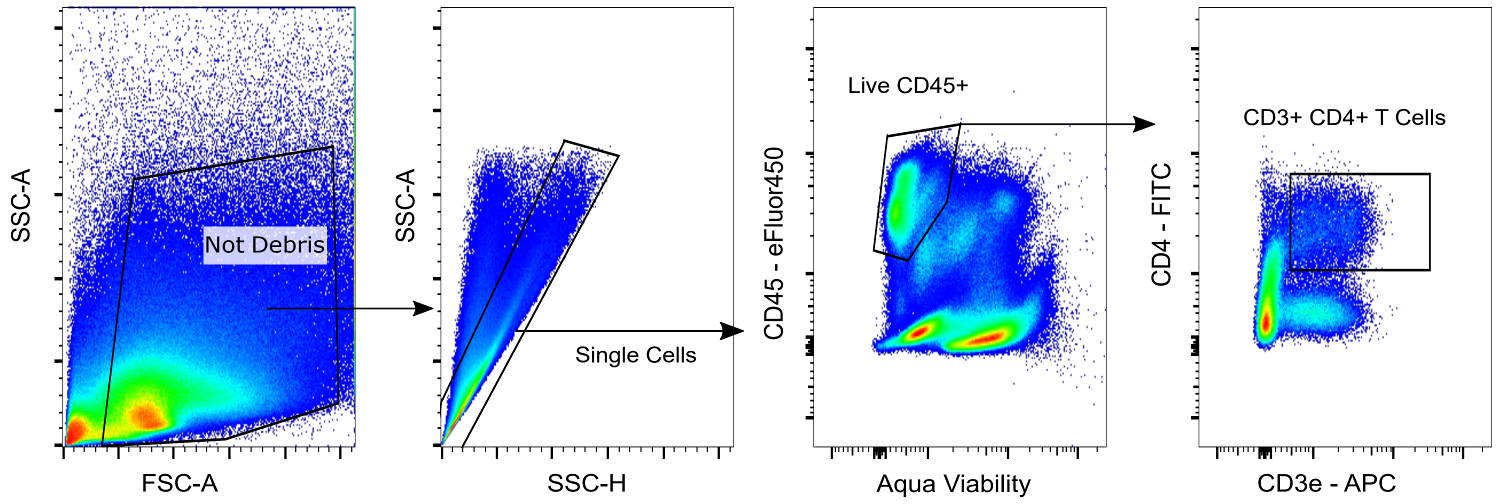

Fig. S4

bioRxiv preprint doi: https://doi.org/10.1101/2023.07.26.550772; this version posted July 27, 2023. The copyright holder for this preprint (which was not certified by peer review) is the author/funder, who has granted bioRxiv a license to display the preprint in perpetuity. It is made available under aCC-BY 4.0 International license.

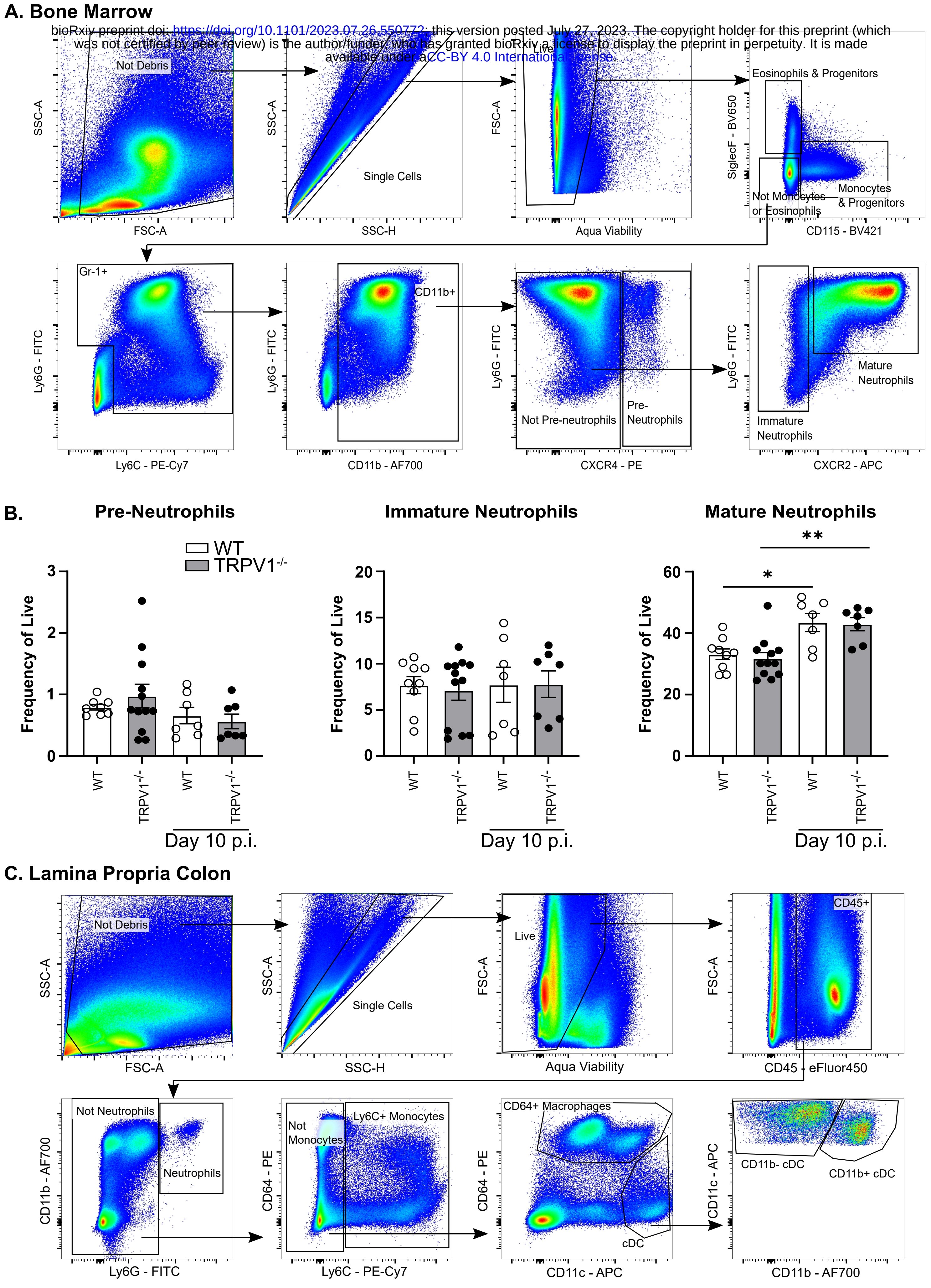

Supplement: 1 [file NIHPP2023.07.26.550772v1-supplement-1.pdf]
